# Boosting Fructosyl Transferase’s Thermostability and Catalytic Performance for Highly Efficient Fructooligosaccharides (FOS) Production

**DOI:** 10.3390/foods13182997

**Published:** 2024-09-21

**Authors:** Dandan Niu, Nan Zhao, Jun Wang, Nokuthula Peace Mchunu, Kugen Permaul, Suren Singh, Zhengxiang Wang

**Affiliations:** 1 Department of Biological Chemical Engineering, College of Chemical Engineering and Material Sciences, Tianjin University of Science and Technology, Tianjin 300457, China; ddniu@tust.edu.cn (D.N.); zn18394004676@163.com (N.Z.); 2 Tianjin Key Laboratory of Industrial Microbiology, College of Biotechnology, Tianjin University of Science and Technology, Tianjin 300457, China; wj8154533@126.com; 3 National Research Foundation, P.O. Box 2600, Pretoria 0001, South Africa; np.mchunu@nrf.ac.za; 4 School of Life Science, University of KwaZulu Natal, Durban 4000, South Africa; 5 Department of Biotechnology and Food Technology, Faculty of Applied Sciences, Durban University of Technology, P.O. Box 1334, Durban 4001, South Africa; kugen@dut.ac.za (K.P.); singhs@dut.ac.za (S.S.)

**Keywords:** high substrate concentration, enzyme-catalyzed FOS synthesis, engineering process optimization, thermostability of enzymes, enzymatic catalysis efficiency

## Abstract

Achieving enzymatic food processing at high substrate concentrations can significantly enhance production efficiency; however, related studies are notably insufficient. This study focused on the enzymatic synthesis of fructooligosaccharides (FOS) at high temperature and high substrate concentration. Results revealed that increased viscosity and limited substrate solubility in high-concentration systems could be alleviated by raising the reaction temperature, provided it aligned with the enzyme’s thermostability. Further analysis of enzyme thermostability in real sucrose solutions demonstrates that the enzyme’s thermostability was remarkedly improved at higher sucrose concentrations, evidenced by a 10.3 °C increase in melting temperature (*T*_m_) in an 800 g/L sucrose solution. Building upon these findings, we developed a novel method for enzymatic FOS synthesis at elevated temperatures and high sucrose concentrations. Compared to existing commercial methods, the initial transglycosylation rate and volumetric productivity for FOS synthesis increased by 155.9% and 113.5%, respectively, at 65 °C in an 800 g/L sucrose solution. This study underscores the pivotal role of substrate concentration, incubation temperature, and the enzyme’s actual status in advancing enzyme-catalyzed processes and demonstrates the potential of enzymatic applications in enhancing food processing technologies, providing innovative strategies for the food industry.

## 1. Introduction

The advent of enzyme catalysis has marked a transformative era in biomanufacturing, offering unparalleled specificity, benign operational conditions, and the facilitation of complex biochemical transformations with remarkable efficiency [1,2]. This paradigm shift has catalyzed the advancement of sustainable and eco-friendly biocatalytic processes [3], as exampled in the synthesis of functional oligosaccharides, where enzymatic methods have overtaken traditional physical and chemical extraction techniques in prevalence and preference [4,5]. Enzymatic catalysis is characterized by a straightforward production process, mild production conditions, high catalytic efficiency, controllable catalytic activity, and high specificity. Currently, the large-scale preparation of functional oligosaccharides through transglycosylation by specific glycosyltransferases is the most significant method for the industrial production of functional oligosaccharides [5].

In the domain of natural ecosystems, the concentration levels of substrates pivotal for enzymatic reactions are subject to significant variability, spanning from micromolar (μM) to millimolar (mM) ranges. This fluctuation is contingent upon the substrate’s nature and its biological context. In certain scenarios, these concentrations may either plummet to nanomolar magnitudes or surge beyond millimolar amounts, dictated by the metabolic environment or ecosystem in focus [6].

Apart from cell-catalyzed synthesis of products, enzyme-catalyzed reactions can be conducted using high substrate concentrations. From the perspective of industrial engineering, the exploitation of enzyme catalysis at high substrate concentrations represents a significant advancement towards enhancing the efficiency and productivity of product synthesis processes [4]. This approach will not only accelerate reaction rates and increase product yields but also promote the efficient use of equipment, while concurrently minimizing reaction volumes and water consumption [7,8]. However, the implementation of reactions at elevated substrate concentrations introduces a series of challenges, including but not limited to issues related to mass transfer, substrate solubility, enzyme inhibition and stability, product extraction, reaction kinetics, and scalability concerns. Among these, mass transfer limitations, which are influenced by factors such as viscosity and mixture homogeneity, as well as issues related to enzyme activity, including inhibition and denaturation, stand out as significant hurdles [9]. A prevalent and efficacious method for enhancing substrate solubility and significantly reducing the viscosity of solutions involves the strategic increase of reaction temperatures [10,11]. This technique effectively addresses the common mass transfer challenges encountered in high-concentration substrate systems. However, this approach introduces additional complexities, particularly in maintaining enzyme activity and its tertiary structural integrity, which are crucial for catalytic functionality [12].

Given that the majority of natural enzyme molecules are inherently sensitive to higher temperature, maintaining their catalytic efficiency at elevated temperatures poses a considerable challenge [13,14]. To address the challenges of limited enzyme thermostability, synthesizing or deriving thermophilic enzymes, characterized by their resilience to high temperatures, is a common recourse. These enzymes are either extracted from thermophilic organisms or engineered as thermotolerant variants through directed evolution techniques [12,15,16,17]. However, not all kinds of enzymes were able to be developed as thermotolerant either through natural selection or directed evolution [18,19,20]. Alternatively, modification of the enzymatic reaction environment has been considered [18,19,20,21,22]. For example, the addition of a certain type of agents, such as ligands, inorganic salts, most polyols, sugars, or surfactants, into the enzymatic reaction mixture might significantly improve the thermostability of enzymes [19,23,24,25,26]. For the effective optimization of these biocatalysts within industrial contexts, it is imperative to meticulously adjust reaction parameters to optimize catalytic efficiency and thermostability [27]. This entails a comprehensive examination of how high substrate concentrations can bolster enzyme stability and the impact of increased temperatures on the viscosity and dynamics of the reaction medium. The ensuing alterations could significantly affect enzyme activity and stability, highlighting the correlation between substrate concentration and the fluidity of the reaction medium under thermal stress as a pivotal research area, with implications for enhancing enzyme-based industrial processes [28,29].

In the presented investigations, the enzyme fructosyl transferase was selected for a detailed examination. This enzyme is crucial in the synthesis of FOS, a category of prebiotics celebrated for their significant health advantages, especially in promoting gastrointestinal wellness [9]. The study aimed to critically analyze the management of enzymatic catalysis processes through the perspective of industrial engineering. This inquiry particularly aimed at deciphering the management of enzymatic reactions at high substrate concentrations, necessitating elevated reaction temperatures. Through this perspective, the characteristics of enzymatic processes were dissected, with a focus on the thermal stability and catalytic efficiency of the enzyme. The outcomes aim to significantly elevate the efficiency of enzymatic catalysis and production from an industrial engineering standpoint. These results harbor the potential to unveil novel insights for innovation in enzyme-catalyzed bio-catalysis engineering and to drive the advancement of related production technologies.

## 2. Materials and Methods

### 2.1. Enzyme and Chemicals

Fructosyl transferase (FTase) preparation (3000 U/mL) from *Aspergillus niger* was ordered from Jiangsu Ruiyang Biotech Co., Ltd. (Wuxi, China). It contains 600 amino acid residues with a predicted molecular weight of 76.5 kD and was prepared for FOS production from sucrose. Monosaccharides and disaccharides references (glucose, sucrose, fructose, fructooligosaccharides, GF_2_, GF_3_, GF_4_, 1-kestose, and galactotriose) were supplied by Sigma-Aldrich (Shanghai, China). All other reagents were of analytical grade.

### 2.2. Enzyme Activity Assay

FTase activity assay was carried out using 100 g/L of sucrose as the substrate in sodium acetate buffer (20 mM, pH 5.5). After the reaction mixture was incubated at 50 °C for 1 h, the samples (0.1 mL) were withdrawn and boiled for 10 min to inactivate the enzyme before HPLC analysis [30]. The enzyme was added at 0.3–3 U/g sucrose to ensure that the activities were determined by the linear part of the progress curve with no detectable fructose formed on HPLC analysis. One unit (U) of the activity is defined as the amount of enzyme that produced 1 µmol 1-kestose per minute under the assay conditions.

### 2.3. Thermal Denaturation Measurements by Fluorescence-Based Thermal Shift Assays (F-TSA)

To investigate the effect of various concentrations of sugars on the unfolding of enzyme proteins, a thermal shift assay using SYPRO Orange dye (Invitrogen, Thermo Fisher Scientific, Waltham, MA, USA) was performed according to the reference with a slight modification [31]. Briefly, 30 µL reactions containing 300 µg/mL enzyme and 5× SYPRO Orange dye without or with various concentrations of sugars were heated from 25 to 95 °C with 1 °C/min incremental increases. An Applied Biosciences Step-One Plus RT-PCR instrument (Applied Biosystems, Carlsbad, CA, USA) equipped with a fixed excitation wavelength (480 nm) and a ROX emission filter (610 nm) was used to monitor the thermal unfolding. All measurements were taken in triplicate.

The unfolding ratio of protein molecules, x*_unf_*, can be calculated [32] as

(1) $$x_{unf}=\frac{f_{T}-f_{N}}{f_{D}-f_{N}}$$

where *f_T_* is the fluorescence intensity at temperature *T*, *f_N_* represents the minimum value of the fluorescence intensity before the transition, and *f_D_* represents the maximum value after the transition. From these curves, the *T*_m_ values of enzyme protein were obtained from the first derivatives of the plot of fluorescence intensities at different temperatures [33].

### 2.4. Thermal Denaturation Measurements by Circular Dichroism (CD) Spectroscopy

Thermal denaturation studies were carried out in a Bio-logic MOS450 spectropolarimeter (Bio-logic, Cecine Parisse, France) equipped with a temperature controller with a heating rate of 1 °C/min. This scan rate was found to provide adequate time for equilibration. Change in CD at 222 nm of the protein solution was measured in the temperature range 45–65 °C. After denaturation, the sample was immediately cooled down to measure the reaction at different temperatures. It was observed that data from renaturation experiments fell on the denaturation curve. All solution blanks showed negligible change in CD with temperature and were, therefore, neglected during the data analysis. The raw CD data were converted into mean residue ellipticity (deg cm^2^ dmol^−1^) at a given wavelength *λ* using the relation

(2) $$mdeg={\left[\theta \right]}_{\lambda }Lc$$

where [θ]*_λ_* is the observed ellipticity (millidegrees) at wavelength *λ*, L is the path length (cm), and c is the protein concentration (mg/cm^3^).

### 2.5. Determination of Water Activity

Water activity (α*_w_*) of sugar solutions was theoretically described by the following equation [34,35].

(3) $$\alpha_{w}=\left(1-X_{S}\right)\exp\left(\alpha X_{s}^{2}+\beta X_{s}^{3}\right)$$

where X*_S_* is the molar fraction of sugar. The experimental parameters, α and β, were determined from the freezing point depression and were obtained from the literature for sucrose, lactose, maltose, glucose, galactose, and fructose [35,36,37].

### 2.6. Estimation of Relative Contribution of Water Activity on Enzyme Conformational Stability

The equilibrium constant between the unfolded and native protein, K*_unf_*, based on the two-state transition [38,39], was described by

(4) $$K_{unf}=\frac{x_{unf}}{1-x_{unf}}$$

Then, the equilibrium between the unfolded and native protein (K*_unf_*) was also described as a function of water activity (α*_w_*) by applying the following Wyman–Tanford equation [39,40,41]:

(5) $$\frac{dlnK_{unf}}{dln\alpha_{w}}=∆n-\left(\frac{X_{w}}{X_{S}}\right)∆m$$

where X*_W_* and X*_S_* are the molar fraction of water and sugar, respectively, Δ*n* is the change of hydration number per protein molecule, and Δ*m* is the change of bound-sugar molecules per protein molecule upon unfolding. The free energy difference, ΔΔG, was measured by calculating the difference values between the free energy for protein unfolding in a solution (ΔG*_D_*_,*S*_) and in water (ΔG*_D_*_,0_). Based on the experimentally proven linear correlation between lnK*_D_* and lnα*_w_*, when α*_w_* = 1, [41] established an equation as follows:

$$\Delta \Delta G=\Delta G_{D,S}-\Delta G_{D,0}=-RT\left(lnK_{D,S}-lnK_{D,0}\right)=-RT\left\{\Delta n-\left(\frac{X_{w}}{X_{S}}\right)\Delta m\right\}\ln\alpha_{w}$$

(6) $$=-RT\left\{\Delta n-\left(\frac{X_{w}}{X_{S}}\right)\Delta m\right\}\left[\ln\left(1-X_{S}\right)+\alpha X_{s}^{2}+\beta X_{s}^{3}\right]$$

where R is the gas constant.

### 2.7. FOS Preparation

To investigate the effects of temperature and substrate concentration on the synthesis of FOS, the reaction kinetics were measured in a shaking water bath at various temperatures (45–75 °C), with 500–800 g/L of sucrose solution (pH 5.5) as the substrate. Reactions with a total volume of 20 mL were initiated by adding 10 units of FTase. The initial transglycosylation reaction rate (*V_c_*, *c* represent substrate concentration) was monitored by the contents of trisaccharides (1-kestose or galactotriose) with tetrasaccharide undetectable within the first 1 h, and the samples (0.5 mL) were boiled for 10 min and analyzed by HPLC.

The comparisons of the FOS preparation in different conditions were further examined in 100 mL working volume with 500 g/L sucrose at 45 °C or 800 g/L sucrose at 65 °C with the same enzyme dosage (3 U/g). The maximum oligosaccharide concentrations were obtained by continuously monitoring the reaction until the concentration of FOS either decreased or did not significantly increase over 1 h. The yield of FOS (%, g/g) was calculated by the proportion of FOS accounted for by the total sugars [42]. Each reaction condition was evaluated in triplicate.

### 2.8. Estimation of Enzymatic Kinetic Synthesis of FOS

The development of the model is based on Michaelis–Menten kinetics, and the initial substrate inhibition factor as an uncompetitive inhibitor was taken into account in the equation [43]. Accordingly, the reaction mechanism was similar to uncompetitive inhibition and was proposed in Equations (7) and (8).

(7) E+S⇋ES→E+P

(8) ES+S⇋SES

where E, S, ES, P, and SES represent the enzyme, initial substrate, enzyme-substrate complex activated state, product, and substrate-enzyme-substrate complex inactivated state, respectively. In this model, Michaelis–Menten equation was shown as follows:

(9) $$V=-\frac{d\left[S\right]}{dt}=\frac{V_{max}\left[S\right]}{K_{m}+\left[S\right]+\frac{{\left[S\right]}^{2}}{K_{si}}}$$

(10) $$\frac{\left[S\right]}{V}=\frac{K_{m}}{V_{max}}+\frac{\left[S\right]}{V_{max}}+\frac{{\left[S\right]}^{2}}{{V_{max}\times K}_{si}}$$

where *V* is the initial reaction rate, *V*_max_ is the maximum reaction rate, *K*_m_ is the Michaelis–Menten constant, *K*_si_ is the uncompetitive inhibition constant by substrate, and [S] is the concentration of the substrate. The kinetic parameter values were determined using MatLab (The MathWorks, Inc., Natick, MA, USA) by nonlinear regression analysis using Equation (10). One set of experimental data on substrate inhibition was chosen for this purpose.

### 2.9. The Sugar Profiles by HPLC Analysis

Quantitative analysis of individual carbohydrates was performed using HPLC (Agilent Technologies, Cheshire, UK) with an ELSD detector 2000 s (Grace Alltech Co., Ltd., MD, USA). The column used was a Prevail™ Carbohydrate ES 5u (250 mm × 4.6 mm, Grace Alltech Co., Ltd., Columbia, MD, USA). A mixture of water/acetonitrile (35:65, *v*/*v*) was used as the mobile phase at a flow rate of 1.0 mL/min. The injection volume was 10 μL. Quantitative measurement of each peak was performed using standard calibration curves of sugar standards as references.

### 2.10. The Statistical Methodology

The experimental data were analyzed using IBM SPSS Statistics v.20 software (IBM Corp., Armonk, NY, USA). The results of tests wherein each data point was derived from the mean of at least three independent experiments were expressed as mean ± standard deviation (SD). A *p*-value < 0.01 was considered to be statistically significant, with significance levels indicated as follows: * *p* < 0.01, ** *p* < 0.01, and *** *p* < 0.001, in accordance with Fisher (1992).

## 3. Results and Discussion

### 3.1. Effect of Temperature on Solubility and Viscosity of Sucrose Solution

**Figure 1:**
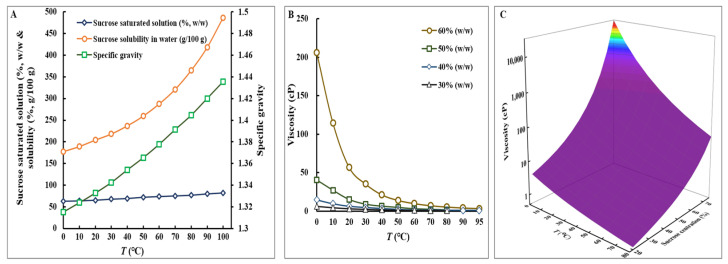
Physiochemical properties of sucrose solutions. (**A**) Solubility of sucrose in water across varying temperatures. Data sourced from Engineering Toolbox https://www.engineeringtoolbox.com/sugar-solubility-water-d_2193.html (accessed on 18 June 2024). (**B**) Viscosity profiles of sucrose solutions at various concentrations and temperatures. Information adapted from Engineering Toolbox “https://www.engineeringtoolbox.com/sugar-solutions-dynamic-viscosity-d_1895.html” (accessed on 18 June 2024). (**C**) The potential relationship between the solubility and viscosity of sucrose solutions at various concentrations and temperatures using data referenced from [45,46].

The dynamic interplay between temperature and the physicochemical properties of sucrose solutions, notably solubility and viscosity, presents a critical focal point in engineering optimization of the production of FOS through the catalytic action of FTase. Therefore, a comprehensive analysis concerning the viscosity variations of sucrose solutions at escalated concentrations and the corresponding solubility alterations across a spectrum of temperatures was carried out, and the results are encapsulated in Figure 1. The solubility of sucrose in water demonstrates a clear dependence on temperature (Figure 1A), a fundamental principle in solution chemistry. At 20 °C, sucrose’s solubility is recorded at 204.07 g per 100 g of water. This solubility progressively increases with temperature. For instance, at 40 °C, the solubility enhances to 236.79 g/100 g water. Notably, at 70 °C, the solubility escalates to 321.03 g/100 g water. The solubility peaks significantly at 100 °C, where it is recorded at an impressive 486.35 g/100 g water. This dataset underscores the pronounced effect of temperature on the solubility of sucrose in water, illustrating a clear trend of increasing solubility with rising temperatures.

On the other side, as the concentration of sucrose in water increases, the viscosity of the solution also rises (Figure 1B). This occurs because a higher concentration of sucrose molecules hinders the flow of the solution, making it thicker and more viscous. Elevated viscosity in high sucrose concentrations results in a diffusion-limited enzyme kinetic microenvironment, where the reaction rate is primarily determined by the rate at which substrate molecules encounter the enzyme’s active site, rather than the enzyme’s catalytic efficiency. According to the Stokes–Einstein equation, increased viscosity reduces the diffusion coefficient of substrate molecules, thereby decreasing their mobility. This reduction in substrate mobility diminishes the frequency of encounters between the substrate and the enzyme, significantly lowering the formation rate of the enzyme-substrate complex and, consequently, the overall reaction rate [44]. On the other hand, as the temperature increases, the viscosity of the sucrose solution decreases. The increase in temperature provides more kinetic energy to the molecules, allowing them to move more freely and thus reducing the resistance to flow, which results in a decrease in viscosity. This inverse relationship between temperature and viscosity is consistent across the entire range of sucrose concentrations from 10% to 60% (*w*/*w*). Therefore, at any given concentration within this range, heating the solution will lower its viscosity, while cooling it will increase its viscosity. Notably, at the zenith of sucrose concentration, nearing saturation levels, the viscosity exhibited a dramatic surge with even marginal increases in concentration (Figure 1C). At a concentration approaching 65% sucrose and maintained at 20 °C, for example, viscosity values are observed to skyrocket, reaching or surpassing 148.2 cP. Conversely, a 60% sucrose solution at 20 °C typically displays a viscosity of approximately 58.93 cP, yet this value can significantly reduce to around 14.0 cP or lower when the temperature is elevated to 50 °C (Figure 1C).

The physicochemical characteristics of a high-concentration sucrose solution conform to the general laws of solutions, and an increase in temperature significantly enhances the solubility of sucrose and notably reduces the viscosity of the sucrose solution. The observed phenomena have profound implications for the enzymatic production of FOS, particularly in terms of optimizing reaction conditions and engineering parameters to enhance the thermostability and catalytic efficiency of FTase. The data underscore the necessity for meticulous temperature control and concentration adjustments to maximize production yields while mitigating viscosity-related and solubility-related challenges in the processing environment.

### 3.2. FTase Tested Is Moderately Heat-Resistant but Sensitive to High Temperatures

**Figure 2:**
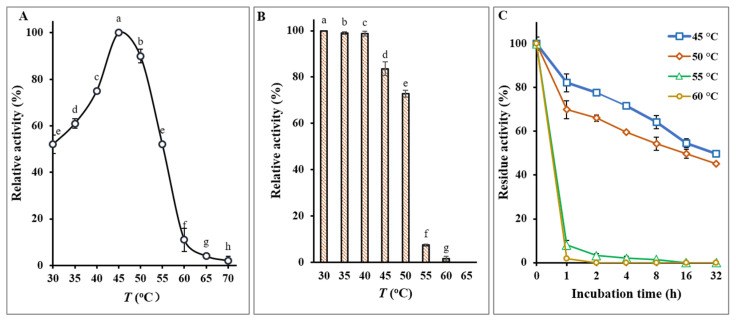
Effects of temperature on FTase activity. (**A**) Examination of the optimal temperature for FTase activity across a range of temperatures. (**B**) Thermostability analysis of the enzyme following incubation at various temperatures for 1 h, with residual activities assessed using the specified enzyme activity assay conducted in triplicate. (**C**) Extended thermostability assessment involving incubation of the enzyme at different temperatures for up to 32 h, followed by determination of residual activities, also performed in triplicate. The letters a–h indicate statistically significant differences in decreasing orders of magnitude (*p* < 0.01).

The above analysis reveals that to meet engineering demands for increasing the concentration of sucrose substrates in the reaction system, it is necessary to simultaneously address the challenge of enhancing sucrose’s solubility while reducing the viscosity of the sucrose solution to ensure subsequent friendly engineering operations. This implies that in the FOS enzymatic production process, the thermal stability of the enzyme preparation must meet the requirements for process changes with temperature increases. For this purpose, in the present study, we have meticulously purified FTase derived from *A. niger* and conducted a comprehensive analysis of its thermostability properties. The enzyme’s optimal activity was observed when acting on a substrate concentration of 100 g/L sucrose, reaching its peak at 45 °C, as depicted in Figure 2A. This finding is consistent with the enzyme’s known preference for moderate temperatures, further substantiating its operational efficacy within this thermal range. A critical examination of the enzyme’s stability under varying temperature conditions revealed a notable resilience at temperatures below 40 °C (Figure 2B). This stability underpins the enzyme’s potential applicability in industrial processes that operate within this temperature spectrum, highlighting its utility in scenarios where moderate heat is applied. However, the enzyme’s stability significantly diminishes when subjected to temperatures in the range of 55–60 °C for a duration of 60 min or longer, with residual activity plummeting to less than 8% of its optimal functionality (Figure 2C). This pronounced decline in activity beyond the 40 °C threshold elucidates the enzyme’s moderate heat resistance, while also underscoring its vulnerability to higher temperatures.

These observations underscore the moderate heat resistance of commercially available FTase, while also highlighting its sensitivity to high temperatures, suggesting that while the enzyme exhibits a degree of resilience to moderate thermal stress, its utility in processes requiring exposure to higher temperatures may be limited. Consequently, these insights into the thermostability of FTase are invaluable for guiding the optimization of industrial processes and for informing the development of enzyme formulations with enhanced thermal stability. Consequently, it comes as no surprise that the current industrial production predominantly employs an enzymatic synthesis temperature of 50 °C for the production of FOS.

### 3.3. Thermal Denaturation Characteristics of FTase in Real Sucrose Solution

**Figure 3:**
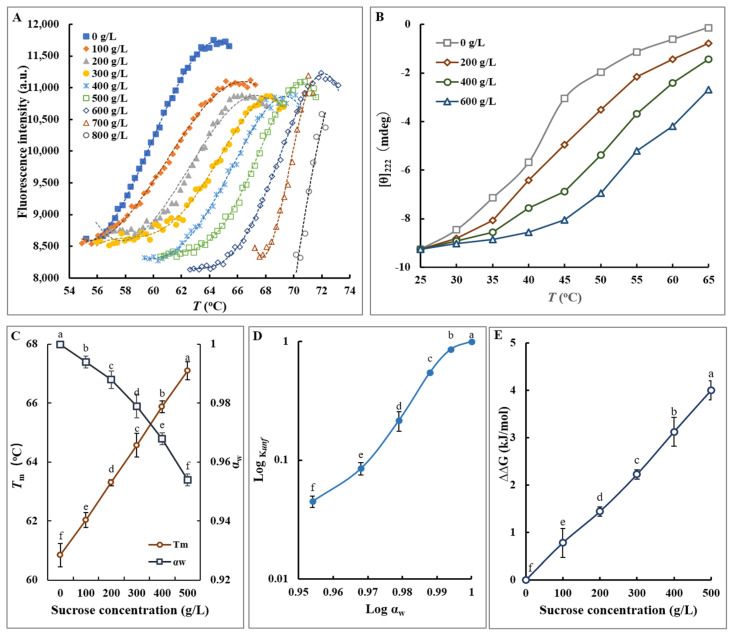
The physicochemical characteristics of denaturation of FTase in sucrose solutions. (**A**) Temperature-fluorescence profiles of FTase in various concentrations of sucrose solutions, monitored using a real-time PCR instrument. Fluorescent dyes were employed to bind to protein structures, enhancing fluorescence signals to compare melting temperatures (*T*_m_) and assess potential structural deformations in the protein. (**B**) Representative thermodenaturation curves of FTase in the presence of different sucrose concentrations, analyzed using circular dichroism. (**C**) Analysis of the relationship between α*_w_* and *T*_m_ in solutions with varying sugar concentrations. (**D**) Determination of the equilibrium constant (K*_unf_*) and α*_w_* in various sugar solutions at different concentrations. (**E**) Correlation between free energy difference (ΔΔG) and sugar concentration. The letters a–f indicate statistically significant differences in decreasing orders of magnitude (*p* < 0.01).

As described in the above text, the commercially available FTase presented a notable gap between its intrinsic thermal stability and the optimal reaction temperatures necessary for advanced engineering applications. Addressing this issue, the present study meticulously examines the thermostability and behavior of FTase in various concentrations of sucrose solutions at different temperatures by determining its melting temperature (*T*_m_), which is a critical parameter indicating the onset of enzyme denaturation [47]. The fluorescence intensity of the FTase solution began to rise at 55 °C and peaked at 64 °C, setting the *T*_m_ of FTase in an aqueous solution at 60.78 °C (Figure 3A). When FTase was incubated in sucrose solutions of increasing concentrations, there was a significant elevation in *T*_m_ values, demonstrating a direct correlation between sucrose concentration and FTase thermal stability (Figure 3A). For example, in an 800 g/L sucrose solution, the *T*_m_ of FTase escalated to 71.08 °C, marking a 10.3 °C increase from its performance in pure water. This trend was consistent across different sucrose concentrations, as also shown by changes in [θ]_222_, indicating alterations in peptide backbone conformation due to heat-induced denaturation (Figure 3B).

Timasheff’s early thermodynamic stabilization mechanism suggests that sucrose as a penetrant varies with the molecular properties of proteins that maximize hydration. During the osmotically regulated reaction, the measured change in the water in contact with the protein is a function of preferential hydration, which means that sucrose enhances protein stability [48]. The thermal unfolding of proteins in solution is a complex interaction involving water and solute molecules, yet the role of water activity (α*_w_*) is often overlooked in thermostability analyses [49]. Further investigation into the impact of sucrose concentration on FTase’s thermal resistance revealed a negative correlation between α*_w_* and *T*_m_, with decreased α*_w_* leading to reduced protein unfolding rates and enhanced thermostability (Figure 3C). When the temperature was fixed at 60 °C for FTase, the plot of lnK*_unf_* to lnα*_w_* showed a linear relationship, as shown in Figure 3D. This relationship showed that decreased αw reduced the unfolding ratio of protein molecules (X*_unf_*) and thus increased the enzyme’s thermostability. This relationship aligns with Miyawaki’s findings on other proteins [50]. Additionally, incubation of FTase with 400 g/L of various sugar solutions increased the ΔΔG (indicative of the change in free energy for protein unfolding) by 2.9–4.9 kJ/mol (Figure 3E), suggesting that higher sugar concentrations increased the ΔG, attributed to the cooperative hydration effect and the concentration-independent Δn and decreased α*_w_* [51]. Consequently, higher sucrose concentrations necessitated a greater ΔG for protein unfolding at elevated temperatures, given the temperature dependence of ΔG [52]. On the other hand, several studies have demonstrated that the formation of the enzyme-substrate complex-activated state stabilizes the tertiary structural of the enzyme under severe reaction conditions [53]. This mechanism should also be present in the research discussed in the present studies. The correlation not only advances our understanding of protein thermostability mechanisms but also highlights the potential for optimizing FTase use in FOS production through strategic sucrose concentration management.

### 3.4. Enhanced Enzymatic Reaction Efficiency in Higher Concentration of Sucrose Solution at Elevated Temperature

**Figure 4:**
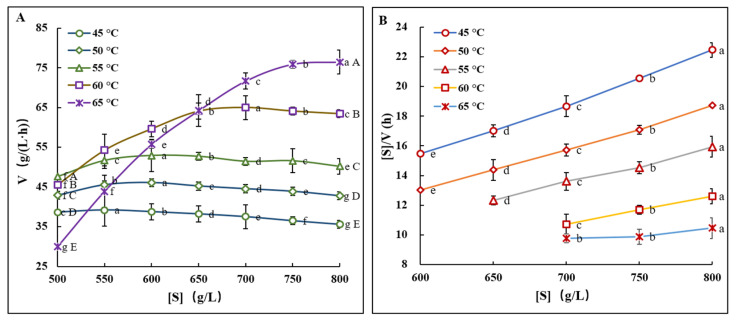
FOS synthesis characteristics with different concentrations of sucrose at different temperatures. (**A**) Initial rate of FOS synthesis. (**B**) The relationship between [S]/V and [S] with substrate inhibition effects. The letters a–g and A–E indicate statistically significant differences in decreasing orders of magnitude (*p* < 0.01).

High substrate concentrations are essential in modern biomanufacturing applications. In this study, the viscosity issues associated with high sucrose concentrations were addressed by increasing the reaction temperature. The results confirmed that high concentrations of sucrose solutions significantly enhance the thermal stability of FTase, thereby protecting and boosting its activity. Consequently, it becomes imperative to ascertain whether FTase, under conditions of elevated temperatures and high sucrose concentrations, maintains its catalytic efficiency for synthesizing FOS, which holds substantial engineering value. Therefore, further examinations were conducted to evaluate the synthetic efficiency of FTase in converting sucrose to FOS, with increased sucrose concentrations and concurrent temperature elevations. The findings, summarized in Figure 4 and Table 1, indicate that the initial rate of FOS synthesis peaked at 39.18 g/(L·h) in a 550 g/L sucrose solution at 45 °C. As the temperature was raised to 50 °C, 55 °C, or 60 °C, the sucrose concentrations for maximum synthesis rates shifted to 600, 650, and 700 g/L, respectively, with corresponding increases in synthesis rates to 46.01, 52.92, and 65.41 g/(L·h). Notably, at 45 °C, the increase of sucrose concentration increased the viscosity in the solution; made the molecules crowded; slowed down the diffusion rate of substrate molecules to the active site of the enzyme, the effective binding rate with substrate molecules, and the change of enzyme conformation; and resulted in a decrease in the formation rate of enzyme-substrate complex and the reaction velocity of FOS [54,55]. However, when the temperature is 65 °C, when the sucrose concentration is increased to 800 g/L, the maximum reaction velocity reaches 76.5 g/(L·h), marking a 155.9% [(76.5 − 29.9)/29.9 × 100%] increase compared to the velocity at a 500 g/L concentration, which was 29.9 g/(L·h) (Figure 4A). This significant enhancement in reaction velocity highlights the positive impact of high sugar concentrations on enzymatic activity at elevated temperatures. Further kinetic analysis (Table 1) revealed that at a reaction temperature of 45 °C, the enzyme demonstrated a Michaelis constant (*K*_m_) of 632.35 g/L. This value decreased as the temperature increased, indicating enhanced enzyme-substrate affinity at elevated temperatures. Concurrently, the substrate inhibition constant (*K*_si_) increased. Notably, the maximum reaction rate (*V*_max_) exhibited a progressive rise from 147.1 g/(L·h) at 45 °C to 156.25 g/(L·h) at 50 °C, 158.73 g/(L·h) at 55 °C, and 175.44 g/(L·h) at 60 °C. The results suggest that FTase maintains robust functionality at high sucrose concentrations within a reasonable increase in reaction temperature.

**Figure 5:**
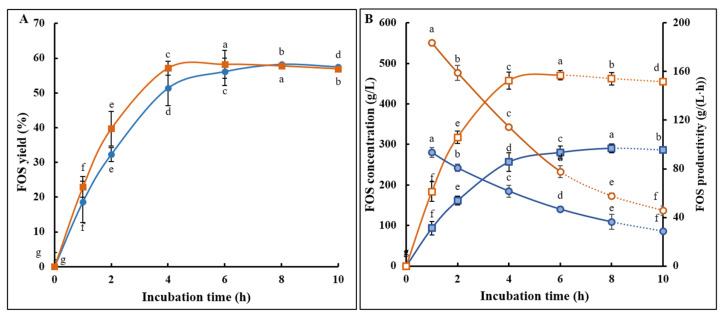
Newly developed process for high-efficiency FOS preparation. (**A**) Comparison of FOS yields at 65 °C with a sucrose concentration of 800 g/L (solid rectangles) versus 45 °C with a sucrose concentration of 500 g/L (solid circles). (**B**) Time-course changes in contents (rectangles) and productivities (circles) during FOS preparation at 65 °C with 800 g/L sucrose (open symbols) versus 45 °C with 500 g/L sucrose (solid symbols). The letters a–g indicate statistically significant differences in decreasing order of magnitude (*p* < 0.01).

These findings are pivotal for both industrial application and further technological advancements in the enzymatic production of FOS. A novel enzymatic method for FOS production was swiftly developed, utilizing a reaction mixture with 800 g/L sucrose at 65 °C. The outcomes have shown significant enhancements in the yield (Figure 5A) and productivity (Figure 5B) of FOS from sucrose. Compared to the existing method of FOS production at 45 °C with 500 g/L of sucrose currently used in industry, the newly developed process increased the FOS yield from 290.9 g/L to 466.1 g/L, achieving a remarkable increase in maximal FOS yield by a 60.2% [(466.1 − 290.9)/290.9 × 100%] rise. It also reduced the reaction time from 8 h to 6 h, a 25% [(8 − 6)/8 × 100%] reduction; FOS productivity increased from 36.4 g/(L·h) to 77.7 g/(L·h), an approximate 1.13-fold [(77.7 − 36.4)/36.4] increase.

On the other hand, an analysis of the different compositions of FOS in the reaction mixture during the FOS production process showed that the FOS produced by the process established in this study did not differ in major compositional makeup from those produced by existing methods (Table 2). The relative proportions were also similar, indicating that the synthesis of FOS at high temperatures and high substrate concentrations achieved in this study primarily enhanced the enzymatic reaction rate without altering the fundamental nature of the enzymatic catalysis.

These results underscore the potential of optimizing enzymatic processes for more efficient FOS production in even higher concentrations of sucrose solution, positioning this novel enzymatic process as markedly superior to existing methodologies in both efficiency and output. The significant uptick in reaction velocity and productivity attributable to this high-efficiency enzymatic process heralds a new potential in functional oligosaccharides preparation, with implications extending beyond FOS production to potentially broader applications in enzymatic catalytic synthesis of other functional oligosaccharides.

## 4. Conclusions

This investigation elucidated that the thermostability of fructosyltransferase was significantly improved in solutions with higher substrate (sucrose) concentrations. Consequently, the enzymatic reaction could be conducted at elevated temperatures and substrate concentrations. As a result, the initial transglycosylation rate and volumetric productivity for FOS synthesis increased by 155.9% and 113.5%, respectively, at 65 °C in an 800 g/L sucrose solution, compared to existing commercial methods. Remarkably, an increase in the enzyme’s catalytic efficiency observed at elevated sucrose concentrations and incubation temperatures paves the way for a novel and highly efficient methodology for FOS synthesis. The findings hold considerable potential for enhancing the optimization of analogous or related enzymatic catalysis processes, thereby contributing significantly to the field of biocatalysis and functional oligosaccharide preparation.

## Figures and Tables

**Table 1:** The kinetic parameters for FOS production at different incubation temperatures.

*T* (°C)	*V*_max_ [g/(L·h)]	*K*_m_ (g/L)	*K*_si_ (g/L)
45	147.1 ± 10.3	632.4 ± 41.5	340.1 ± 19.3
50	156.3 ± 14.6	567.2 ± 25.0	426.7 ± 34.7
55	158.7 ± 31.8	507.9 ± 36.1	525.1 ± 55.3
60	175.4 ± 26.3	447.4 ± 34.7	712.5 ± 68.6

**Table 2:** The characteristics of sugar profiles in FOS syrup prepared by newly developed process.

Process	Incubation Time (h)	Concentration (g/L) and Percentage (%) of FOS Profiles *			
		GF_2_	GF_3_	GF_4_	Total FOS
With 45 °C, 500 g/L	8	170.28 (34.06)	112.06 (22.41)	8.51 (1.70)	290.86 (58.17)
	10	152.09 (30.42)	127.80 (25.56)	10.91 (2.18)	290.80 (58.16)
	12	142.58 (28.52)	134.46 (26.89)	12.39 (2.48)	289.44 (57.89)
With 65 °C, 800 g/L	8	261.20 (32.65)	181.40 (22.68)	17.89 (2.24)	460.49 (57.56)
	10	236.52 (29.57)	194.99 (24.37)	22.61 (2.83)	454.12 (56.77)
	12	218.64 (27.33)	202.94 (25.37)	27.10 (3.39)	448.68 (56.09)

* The data without brackets represented the actual concentration of each component in FOS syrup, measured in g/L. The data in brackets indicated the percentage of each component relative to the total sugar content, measured in %.

## Data Availability

The original contributions presented in the study are included in the article; further inquiries can be directed to the corresponding author.
